# An ABA-responsive DRE-binding protein gene from *Setaria italica*, *SiARDP*, the target gene of SiAREB, plays a critical role under drought stress

**DOI:** 10.1093/jxb/eru302

**Published:** 2014-07-28

**Authors:** Cong Li, Jing Yue, Xiaowei Wu, Cong Xu, Jingjuan Yu

**Affiliations:** State Key Laboratory of Agrobiotechnology, College of Biological Sciences, China Agricultural University, Beijing 100193, China

**Keywords:** Abscisic acid (ABA), abiotic stress, dehydration-responsive element (DRE), foxtail millet, SiARDP1, SiAREB, signal pathway, transcription factor.

## Abstract

The DREB (dehydration-responsive element binding)-type transcription factors regulate the expression of stress-inducible genes by binding the DRE/CRT *cis*-elements in promoter regions. The upstream transcription factors that regulate the transcription of DREB transcription factors have not been clearly defined, although the function of DREB transcription factors in abiotic stress is known. In this study, an abscisic acid (ABA)-responsive DREB-binding protein gene (*SiARDP*) was cloned from foxtail millet (*Setaria italica*). The transcript level of *SiARDP* increased not only after drought, high salt, and low temperature stresses, but also after an ABA treatment in foxtail millet seedlings. Two ABA-responsive elements (ABRE1: ACGTGTC; ABRE2: ACGTGGC) exist in the promoter of *SiARDP*. Further analyses showed that two ABA-responsive element binding (AREB)-type transcription factors, SiAREB1 and SiAREB2, could physically bind to the ABRE core element *in vitro* and *in vivo*. The constitutive expression of *SiARDP* in *Arabidopsis thaliana* enhanced drought and salt tolerance during seed germination and seedling development, and overexpression of *SiARDP* in foxtail millet improved drought tolerance. The expression levels of target genes of *SiARDP* were upregulated in transgenic *Arabidopsis* and foxtail millet. These results reveal that *SiARDP*, one of the target genes of SiAREB, is involved in ABA-dependent signal pathways and plays a critical role in the abiotic stress response in plants.

## Highlight text

SiARDP is a DREB-type transcription factor from foxtail millet. SiARDP is involved in ABA-dependent signal pathways under the control of SiARDP and plays a positive role in plant responses to drought stress.

## Introduction

The growth of plants and productivity of crops are limited by environmental stresses, such as drought, high salinity, soils, and low temperatures. To respond and adapt to these stresses, a large number of specific genes are induced, such as molecular chaperones, osmotic adjustment proteins (Tamura *et al.*, 2003), ion channels (Ward and Schroeder, 1994) and others (Ingram and Bartels, 1996; Thomashow, 1999). Most of these functional proteins are regulated by specific transcription factors (Zhu, 2002; Chinnusamy *et al.*, 2004; Bartels and Sunkar, 2005; Yamaguchi-Shinozaki and Shinozaki, 2006).

Abscisic acid (ABA)-responsive element binding (AREB) transcription factors are members of the group A subfamily of the bZIP transcription factor family and play a key role in ABA-responsive abiotic stress (Jakoby *et al.*, 2002; Correa *et al.*, 2008). AREB proteins bind to ABA-responsive elements (ABREs), which are major *cis*-elements in the ABA-responsive gene promoter region (Giraudat *et al.*, 1994; Busk and Pages, 1998). The AREB transcription factors respond mainly to drought and high salinity stresses and are involved in the regulation of gene expression in the ABA-dependent signal transduction pathway (Finkelstein *et al.*, 2002; Fujita *et al.*, 2005).

The APETALA2 (AP2)/ethylene-responsive factor (ERF) proteins constitute a transcription factor family, and there are 145 ERF/AP2 members in *Arabidopsis thaliana* (Sakuma *et al.*, 2002). Dehydration-responsive element binding (DREB) transcription factors, a subfamily of the ERF/AP2 family, were first isolated using yeast one-hybrid screening of *Arabidopsis* cDNA (Stockinger *et al.*, 1997; Liu *et al.*, 1998). The proteins bind to an 8-bp conserved sequence (ACCGACA), named the dehydration responsive element (DRE), in the *rd29A* promoter (Yamaguchi-Shinozaki and Shinozaki, 1994). Since then many DREB-type transcription factors have been cloned and identified, and most have been confirmed to enhance abiotic stress tolerance in plants (Dubouzet *et al.*, 2003; Qin *et al.*, 2007; Agarwal *et al.*, 2010). DREB transcription factors *DREB1* and *DREB2* are involved in different stress response pathways. The *DREB1* genes were rapidly induced by cold stress and activated the expression of their target genes (Jaglo-Ottosen *et al.*, 1998; Kasuga *et al.*, 1999). Overexpression of *DREB1* enhanced transgenic *Arabidopsis* tolerance to cold, dehydration, and salt stress, and induced accumulation of osmoprotectants, such as proline and various sugars (Gilmour *et al.*, 2000). Several *DREB1*-homologous genes, *OsDREB1* and *OsDREB2* from rice, *HvCBF2* and *HvCBF* from barley (*Hordeum vulgare*), *ZmDREB1A* from maize (*Zea mays*), and *PgDREB2A* from sorghum, have been identified (Dubouzet *et al.*, 2003; Xue, 2003; Qin *et al.*, 2004; Skinner *et al.*, 2005; Agarwal *et al.*, 2010). The *DREB2* genes were induced by osmotic stresses (Liu *et al.*, 1998). However, overexpression of *DREB2A* did not increase stress tolerance in transgenic *Arabidopsis*. A domain analysis showed that DREB2A contains a negative regulatory domain. An active form of DREB2A (DREB2A-CA), in which the negative regulatory region was deleted, could improve drought tolerance and activate the expression of the target genes in transgenic *Arabidopsis* (Sakuma *et al.*, 2006). Most *DREB2* genes were induced by drought and high salinity. *ZmDREB2A*, *TaDREB1*, and *PgDREB2* also respond to low temperature, and *ZmDREB2A* was also induced by high temperature (Shen *et al.*, 2003; Qin *et al.*, 2004; Egawa *et al.*, 2006).

The ABA-independent and ABA-dependent signal transduction pathways are the main abiotic stress response pathways in plants (Yamaguchi-Shinozaki and Shinozaki, 2005). Most of the DREB transcription factors are reported to be involved in the ABA-independent pathway; however, a few are responsive to ABA signalling, such as *DBF1*, *CBF4*, and *OsDREB1F* (Kizis and Pagès, 2002; Haake *et al.*, 2002; Wang *et al.*, 2008). Currently, few reports on the mechanisms involved in the upstream regulation of ABA-induced DREB-type transcription factor genes have been published.

Foxtail millet (*Setaria italica*), an ancient crop in China, provides rich nutrient elements and has excellent drought tolerance (Barton *et al.*, 2009). It grows in arid or marginal soils and is of great significance in developing countries (Bettinger *et al.*, 2010). Being a close relative to major food crops and bioenergy grasses, and having a small diploid genome (~510Mb), it has become an ideal model species (Doust *et al.*, 2009; Lata *et al.*, 2012). Recently, the reference genome sequence and genome-wide association studies (GWAS) of diverse foxtail millet varieties have been generated (Jia *et al.*, 2011; Bennetzen *et al.*, 2012; Zhang *et al.*, 2012). Because of its excellent drought tolerance and water-use efficiency, research on the mechanisms of drought tolerance of foxtail millet is very important. In the present study, an ABA-responsive DREB-like protein gene, *SiARDP*, was cloned from foxtail millet cDNA using a yeast one-hybrid screening assay. The transcription levels of *SiARDP* increased under exogenous ABA treatment, as well as under drought and high salt stress. We cloned and identified two ABA-responsive AREB transcription factor genes from foxtail millet. *In vitro* and *in vivo* assays showed that these two AREB transcription factors could bind to the ABRE elements in the promoter region of *SiARDP*. Meanwhile, we examined the functions of *SiARDP* in *Arabidopsis* and foxtail millet, and found SiARDP was an important regulator for abiotic stress responses during seed germination and seedling development. The results show that in foxtail millet *SiARDP* might be involved in different signalling pathways, and two AREB proteins could be involved in the regulation of *SiARDP*.

## Materials and methods

### Plant materials and treatments

Seeds of foxtail millet (*Setaria italica*, cv. Jigu 11) were germinated on moist gauze for 24h at 28°C, and then grown in pots filled with nutrient soil and vermiculite mixed 1:1 (v/v) under a 16 h:8h (light:dark cycle) at 28°C and 60% relative humidity for 2 weeks. Then, the soil and vermiculite attached to the seedling roots were washed away. The seedlings were fixed in plastic foam, transferred to 1/3 Hoagland solution and grown hydroponically at 26°C for 3 d. They were then subjected to various treatments. During culture, the Hoagland solution was changed every day, and an aeration system was used. For salt, dehydration, and ABA treatments, the seedling roots were immersed separately in 1/3 Hoagland solution containing 100mM NaCl, 20% PEG-6000 and 10 μM ABA, respectively, and kept for the time indicated. For the cold treatment, 17-day-old seedlings grown in soil were maintained at 4°C for the time indicated. Meanwhile, seedlings cultured in 1/3 Hoagland solution without treatment for the corresponding times indicated were used as controls.

### Yeast one-hybrid assay

All of the bait and mutant bait sequences were inserted into the pAbAi vector at the HindIII and XhoI sites to create the bait vectors. The bait vectors were transformed into yeast strain Y1HGold following the protocol of the Yeastmaker™ Yeast Transformation System 2 (Clontech, USA). The bait strains were screened on synthetic defined (SD) medium lacking uracil and containing different concentrations of Aureobasidin A (AbA).

The foxtail millet cDNA library was constructed following the protocol of the Matchmaker™ Gold Yeast One-Hybrid Library Screening System (Clontech, USA).

*SiARDP, SiAREB1* and *SiAREB2* were cloned into pGADT7-AD at the NdeI and XhoI sites as prey vectors. They were transformed into bait strains and grown on SD medium lacking leucine and containing 800ng ml^−1^ AbA for 3 d at 30°C.

### RNA extraction and RNA analysis

Total RNA was isolated using TRIzol reagent (Invitrogen, USA). After digestion with RNase-free DNase I (Takara, Japan), ~2 μg of total RNA was used for reverse transcription by M-MLV Reverse Transcriptase (Promega, USA).

Reverse transcription polymerase chain reaction (RT-PCR) was performed using 2 × Taq PCR StarMix with Loading Dye (GenStar, China). PCR conditions were 95°C for 3min, followed by 24 cycles of 95°C for 30 s, 60°C for 30 s, 72°C for 30 s, and 72°C for 5min. Quantitative RT-PCR (qRT-PCR) assays were performed with a LightCycler 480 II real-time PCR detection system (Roche, USA) using the UltraSYBR Mixture (CWBIO, China). The PCR conditions were 95°C for 10min, followed by 40 cycles of 95°C for 15 s, and 57°C or 60°C for 1min. The ΔΔC_T_ method was used to calculate the expression levels of relevant genes.

### Plasmid construction for subcellular localization analysis

The full-length sequences of *SiARDP*, *SiAREB1* and *SiAREB2* without the stop codons were inserted into a modified pUC-GFP plasmid. The AT-hook motif nuclear-localized protein 22 gene, *AHL22* (BR000358), without the stop codon, was cloned into a modified pUC-RFP plasmid as the positive control. The primers used are shown in Supplementary Table S2 at JXB online. The genes were driven by the cauliflower mosaic virus (CaMV) 35S promoter. Each plasmid (20 μg) was transformed into millet protoplasts. After culturing for 16 to 20h, fluorescence was observed under a confocal laser microscope (Leica HQ).

### Protoplast isolation and transfection

Seeds of foxtail millet were germinated in pots filled with nutrient soil and vermiculite mixed 1:1 (v/v) under 12 h:12h (light:dark) conditions at 26°C for 3 d, and then were moved to the dark for another 4–6 d. Tissues from the stems and leaves were used. A bundle of foxtail millet plants was cut into 0.5–1mm wide strips. Protoplast isolation and transfection were carried out according to the procedure described by Zhang *et al.* (2011). Finally, the protoplasts were resuspended gently in 1ml W5 solution (154mM NaCl, 125mM CaCl_2_, 5mM KCl, and 2mM MES at pH 5.7) and cultured in the dark at 26°C for 16h.

### Transcriptional activation in yeast assay

The open reading frames of *SiARDP*, *SiAREB1*, and *SiAREB2* were inserted into the pBD-GAL4 plasmid (Stratagene, USA) at the EcoRI and SalI sites under the control of the yeast alcohol dehydrogenase 1 (*ADH1*) promoter. The pBD-GAL4 plasmid was used as the negative control and the pGAL4 vector as the positive control. These plasmids were independently transformed into the yeast YRG-2 strain. These transformed yeast cells were grown on SD medium lacking threonine, or lacking threonine and histidine, for 3 d at 30°C.

### Electrophoretic mobility shift assay (EMSA)

**Fig. 1.**
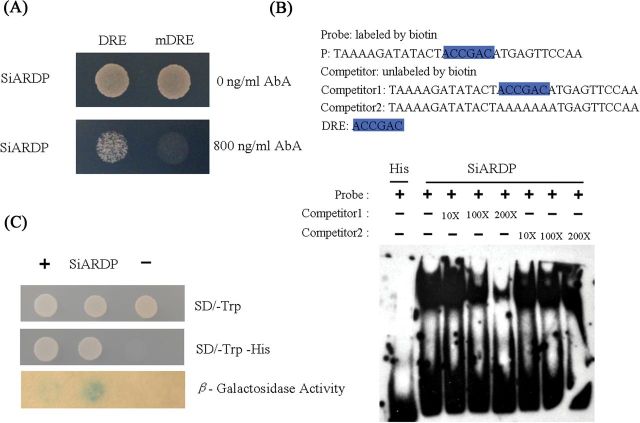
DNA binding ability and transcriptional activation assay of SiARDP. (A) Yeast one-hybrid assay in which SiARDP binds to the DRE core element. The bait sequences are shown in Supplementary Table S2 at *JXB* online. (B) SiARDP bound to various elements. The DRE (highlighted) was labelled with these probe sequences. (C) Transcriptional activation assay of *SiARDP.* The plus symbol indicates that the yeast was transformed with the pGAL4 plasmid as the positive control. The minus symbol indicates that the yeast was transformed with the pBD-GAL4 plasmid as the negative control. This figure is available in colour at *JXB* online.

*SiARDP* was cloned into the pET-28a vector, which contained a Flag tag, at the NdeI and XhoI sites, while *SiAREB1* and *SiAREB2* were inserted into the NdeI and XhoI sites of the modified pGEX-TEV vector containing a GST tag. These fusion proteins were expressed in *Escherichia coli* (BL21) and purified using nickel NTA (Qiagen, Germany) and Glutathione Sepharose 4B (GE, USA), respectively. Oligonucleotides and their reverse complementary oligonucleotides, which were labelled with biotin, were synthesized. These sequences are shown in Figs 1B and 7B. Double-stranded DNA was obtained by heating oligonucleotides at 92°C for 30 s, and annealing at 30°C. The gel-shift assay was performed following the manufacturer’s protocol for the LightShift^®^ Chemiluminescent EMSA Kit (Thermo, USA).

### Generation of *SiARDP* transgenic *Arabidopsis* plants

The full-length sequence of *SiARDP* was amplified by RT-PCR and inserted into the modified binary vector pS1300 at the HindIII and SpeI sites, and was driven by the CaMV 35S promoter. The plasmid was transformed into *Arabidopsis* Col-0 by the vacuum infiltration method (Bechtold and Pelletier, 1998). The seeds of transformed *Arabidopsis* were screened on Murashige and Skoog medium containing 50 μg ml^−1^ hygromycin. Three independent lines of the T3 generation were chosen by the expression levels of *SiARDP* for further analysis.

### Foxtail millet transformation and regeneration

*SiARDP* was cloned into the BamHI and SacI sites of the modified binary vector pCoU under the control of the rice (*Oryza sativa*) ubiquitin promoter, and the vector was introduced into *Agrobacterium tumefaciens* strain LBA4404. The transformation was performed according to the method reported by Wang *et al.* (2011).The calli were screened on medium containing 10 μg ml^−1^ hygromycin for 4 weeks. The transgenic plants were examined by PCR for the presence of hygromycin phosphotransferase II and by western blotting. Two independent lines of the T2 generation were chosen for further analysis. The expression levels of *SiARDP* in the transgenic millet were determined by qRT-PCR.

### Stress treatments of transgenic *Arabidopsis* and foxtail millet

Approximately 70 seeds from the wild-type (WT) and each selected line of the T3 generation of transgenic *Arabidopsis* were used for the phenotypic analysis. For the high salinity treatment, seeds of WT and transgenic *Arabidopsis* were sown on the MS medium containing 0, 100, 150, and 175mM NaCl for 8 d at 22°C, and then the fresh/dry weights of the WT and transgenic *Arabidopsis* were measured. Additionally, 35-day-old seedlings from WT and transgenic *Arabidopsis* grown on MS medium were transplanted on MS medium containing 0, 150, 200, and 250mM NaCl for 4 d at 22°C.

For the dehydration treatment, ~70 seeds from WT and transgenic *Arabidopsis* were sown on MS medium containing 0, 200, and 300mM mannitol for 8 d at 22°C, and then the fresh/dry weights of the WT and transgenic *Arabidopsis* were measured. To test drought tolerance, 30 seeds each from WT and transgenic *Arabidopsis* were grown on MS medium for 1 week and transferred to pots filled with soil and vermiculite (1:1, v/v) for an additional 2 weeks at 22°C and 60% relative humidity. Water was withheld for 14 d as the control condition. The survival rate was counted 5 d after rewatering. Approximately 48 seeds from WT and transgenic millet were germinated on moist gauze for 24h at 28°C and then transferred into pots filled with soil and vermiculite (1:1, v/v) for an additional 2 weeks under conditions of 28°C day:25°C night. Two-week-old seedlings were not watered for 14 d, and then were rewatered and grown under normal conditions for 5 d. The survival rate was calculated.

Relative electrolyte leakage rates and proline contents were measured as described by Zhao *et al.* (2009).

The survival rate, fresh/dry weights, relative electrolyte leakage rate, and proline content data were subjected to Student’s t-test analyses using GraphPad Prism 5. All of the experiments were repeated three times.

### SiARDP-regulated gene expression analysis

Total RNA of non-transgenic *Arabidopsis* and transgenic *Arabidopsis* were used to examine the expression levels of *Rd29A*, *Rd29B*, *Rd17*, and *MT2A* by qRT-PCR. The primers are shown in Supplementary Table S2 at JXB online.

Total RNA was isolated from non-transgenic millet and transgenic millet, and was used to examine the expression levels of stress-relevant genes (Supplementary Table S1 at JXB online). The primers are shown in Supplementary Table S2 at JXB online.

### Chromatin immunoprecipitation (ChIP) assay

Approximately one million foxtail millet protoplasts were transfected with *35S:SiAREB1-GFP* or *35S:SiAREB2-GFP*. Then, the protoplasts were incubated at 26°C in the dark for 16h. The harvested protoplasts were resuspended in a W5 solution containing 1% formaldehyde and crosslinked for 20min. ChIP was performed using the EpiQuik Plant ChIP Kit (Epigentek, Germany) according to the instructions of the manufacturer. The anti-GFP antibody (Sigma, USA) was used. To calculate the enrichment, the C_T_ values were normalized against the input C_T_, where ΔC_T_ = CT (sample) − CT (input). The primer sequences are listed in Supplementary Table S2 at JXB online.

## Results

### Isolation and identification of *SiARDP*

To isolate cDNAs encoding DRE binding proteins from foxtail millet, the one-hybrid library screening system was used. A triplicate 16-bp DNA fragment (ATACTACCGACATGAG) between positions −156 and −171 in the *rd29A* promoter, which contains a DRE core sequence (ACCGAC) at its centre, was cloned into pAbAi as a bait plasmid. A triplicate 16-bp mutant DNA fragment in which the DRE core sequence ACCGAC was substituted with AAAAAA was used as a negative control. The bait plasmid and control were independently transformed into yeast. These transformed yeast cells could grow on media lacking uracil but could not grow on media containing 800ng ml^−1^ Aureobasidin A (AbA) (data not shown). Thus, the concentration of 800ng ml^−1^ AbA was chosen as a screening criterion, and more than 2.0×10^6^ yeast transformants from a library prepared using 2-h and 6-h dehydrated foxtail millet were screened. A millet cDNA encoding a protein with a highly conserved ERF/AP2 DNA-binding domain and two nuclear localization signals (Supplementary Figure S1A and B at JXB online) was cloned. The yeast one-hybrid assay was conducted again to confirm the results. The cDNA was cloned into the yeast expression vector pGADT7-AD. The recombinant plasmid was separately transformed into the bait and mutant bait strains. The transformed bait yeast cells grew on medium lacking leucine and containing 800ng ml^−1^ AbA, but the transformed mutant bait yeast cells did not (Fig. 1A). The cloned cDNA showed high homology with the grass DREB-type transcription factors, such as *PgDREB2A* and *ZmDREB2A* (Supplementary Figure S1C at JXB online), indicating that the transcription factor belonged to the DREB2 family. It was named *SiARDP*.

To confirm the yeast one-hybrid results, an EMSA was performed. SiARDP was expressed as a Flag-tagged fusion protein in *E.coli*. Because DREB transcription factors bind to the DRE core element ACCGAC in the *rd29A* promoter (Liu *et al.*, 1998), the sequence was used as a probe (Fig. 1B). The results are shown in Fig. 1B. The SiARDP fusion protein bound the probe, and the signal was gradually diminished by the addition of the unlabelled DNA probe but not by the addition of the mutant unlabelled DNA probe.

Additionally, the ability of SiARDP to activate transcription was examined in yeast. The full-length *SiARDP* sequence was inserted into pBD-GAL4, and *SiARDP* was fused with the DNA sequence encoding the GAL4 DNA-binding domain. The fusion gene was driven by the yeast *ADH1* promoter. The yeast cells carrying the pBD-SiARDP plasmid grew on medium lacking threonine and histidine. Compared with the GAL4 negative control, SiARDP strongly activated the histidine reporter gene and β-galactosidase activity (Fig. 1C). The result indicates that full-length SiARDP can activate transcription.

### *SiARDP* gene expression and protein localization

**Fig. 2.**
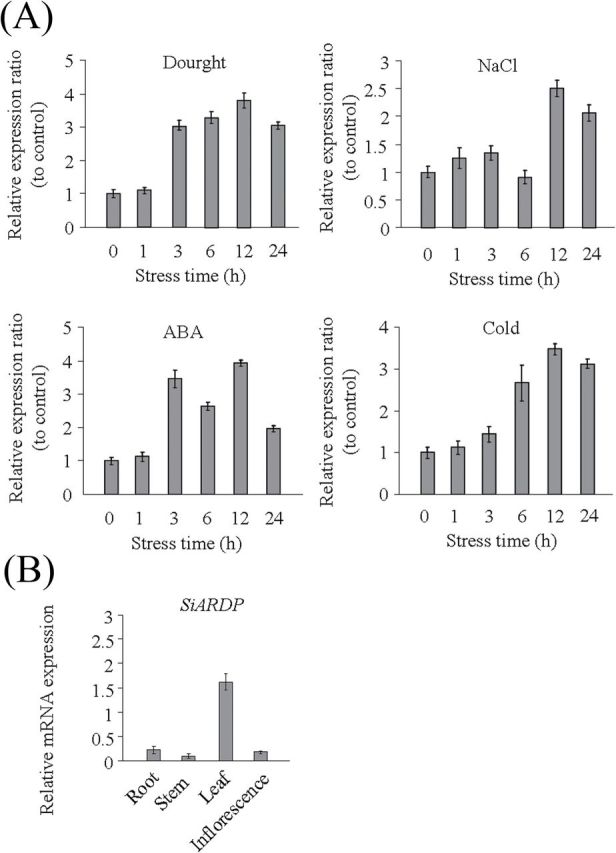
Expression pattern of the *SiARDP* gene. (A) The relevant expression ratios of *SiARDP* (the relevant expression level of *SiARDP* in the treatment/the relevant expression level of *SiARDP* in the untreated control) in response to various stresses in foxtail millet seedlings as demonstrated by qRT-PCR. The millet seedlings were treated under NaCl (100mM), PEG (20% V/V), ABA (10 μM) and 4°C for 0, 1, 3, 6, 12, and 24h, and untreated seedlings at the corresponding time points were used as controls. (B) Transcript levels of SiARDP in different tissues of WT foxtail millet. Foxtail millet actin (AF288226) was amplified as a normalization control.

The expression patterns of *SiARDP* under abiotic stresses were analysed using qRT-PCR. The seedlings were treated with different stresses for 0, 1, 3, 6, 12, and 24h, and untreated seedlings at the corresponding time points were used as controls. The relevant expression ratios (the relevant expression level of *SiARDP* in the treatment/the relevant expression level of *SiARDP* in the untreated control at the same time point) of each time point were calculated. The results showed that the expression of *SiARDP* increased during drought, low temperature, and high salinity treatments. *SiARDP* expression was also induced by ABA treatment. The transcript level of *SiARDP* was obviously induced after 3h of ABA treatment and reached its highest level at 12h. The amount of *SiARDP* showed a significant decrease after the 24-h treatment (Fig. 2A). These results imply that *SiARDP* may be involved in different stress responses. The expression levels of *SiARDP* in different foxtail millet organs were also examined. The transcript levels of *SiARDP* were highest in leaf, and lower in root, stem, and inflorescence (Fig. 2B).

**Fig. 3.**
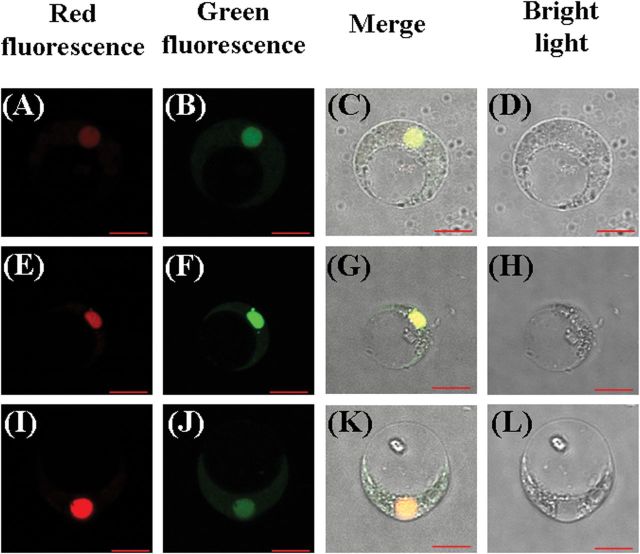
Subcellular location of SiARDP, SiAREB1, and SiAREB2. (A–D) The co-localization of SiARDP-GFP with AHL22-RFP in the nuclei of millet protoplasts. (E–H) The co-localization of SiAREB1-GFP with AHL22-RFP in the nuclei of millet protoplasts. (I–L) The co-localization of SiAREB2-GFP with AHL22-RFP in the nuclei of millet protoplasts. Scale bar: 10 μm. This figure is available in colour at *JXB* online.

We examined the subcellular localization of *SiARDP* and *SiARDP* fused to GFP, as well as *AHL22* fused to RFP as a positive control. The fusion genes driven by the CaMV 35S promoter were transiently expressed in foxtail millet protoplasts. The results indicate that SiARDP was located in the nucleus (Fig. 3A–D).

### Overexpression of *SiARDP* enhances abiotic stress tolerance of transgenic *Arabidopsis*

**Fig. 4.**
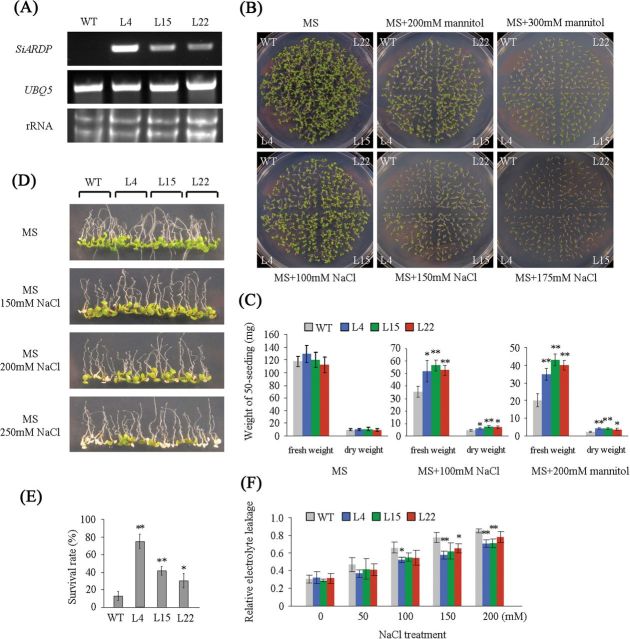
Overexpression of *SiARDP* improves dehydration and salt stress tolerance in *Arabidopsis*. (A) Expression levels of *SiARDP* in transgenic *Arabidopsis* and WT plants (negative control). (B) NaCl or mannitol sensitivity of WT and transgenic plants. Seeds of WT and transgenic lines were germinated and grown for 8 d on medium containing 0 (control), 200, and 300mM mannitol or 100, 150, and 175mM NaCl. (C) Fresh/dry weights of 8-day-old seedlings grown under normal conditions, 100mM NaCl, and 200mM mannitol. All samples were measured in triplicate. (D) NaCl tolerance of WT and transgenic lines. WT and transgenic plants were germinated on MS medium for 5 d, and then transferred to MS medium supplemented with different concentrations of NaCl for 4 d. (E) Survival rate of plants in (D) under salt stress. This experiment had three replicates, and each experiment comprised at least 30 plants. (F) Relative electrolyte leakage in WT and transgenic lines after salt stress. Each data point had three replicates. For C, E, and F, the error bars indicate ±SD, and * and ** indicate statistically significant differences at *P* < 0.05 and *P* < 0.01 (Student’s t-test), respectively. This figure is available in colour at *JXB* online.

To analyse the function of *SiARDP*, it was overexpressed under the control of the CaMV 35S promoter in *Arabidopsis*. At least 30 transgenic *Arabidopsis* plants were obtained using a vacuum infiltration method (Bechtold *et al.*, 1998), and three independent homozygous T3 generation lines with relatively high expression levels of the transgene (Fig. 4A) were used for further investigations.

*SiARDP* was responsive to high salinity, low temperature, and dehydration stress; therefore, our study focused on the abiotic stress tolerance of transgenic *Arabidopsis*. To analyse the influence of salt and dehydration stress on seed germination and growth, the seeds of non-transgenic and transgenic *Arabidopsis* were germinated on MS medium containing different concentrations of NaCl or mannitol. Almost all of the seeds germinated on the medium, but the seedling growth of transgenic *Arabidopsis* overexpressing *SiARDP* and non-transgenic *Arabidopsis* was different under different abiotic stress conditions. No obvious difference was observed between the transgenic and non-transgenic plants under normal conditions. However, the *SiARDP-*overexpressing transgenic seedlings were more tolerant than the non-transgenic seedlings under 100, 150, and 175mM NaCl or 200 and 300mM mannitol stress conditions (Fig. 4B). The fresh/dry weight of seedlings showed that the influence of the abiotic stress on the transgenic seedlings was weaker than on the non-transgenic plants (Fig. 4C).

To further determine the effect of *SiARDP* overexpression on high salinity tolerance, 5-day-old plants growing on a normal medium were transferred to media containing different concentrations of NaCl and maintained for 5 d. Under normal conditions, no obvious differences were observed between WT and transgenic seedlings. The survival rates of the three *SiARDP* transgenic lines growing on a medium containing 250mM NaCl were significantly higher than that of WT plants (Fig. 4D and E). Electrolyte leakage assays showed that the level of ion leakage in the transgenic plants was lower than that in WT plants, especially under high salinity stress (Fig. 4F).

**Fig. 5.**
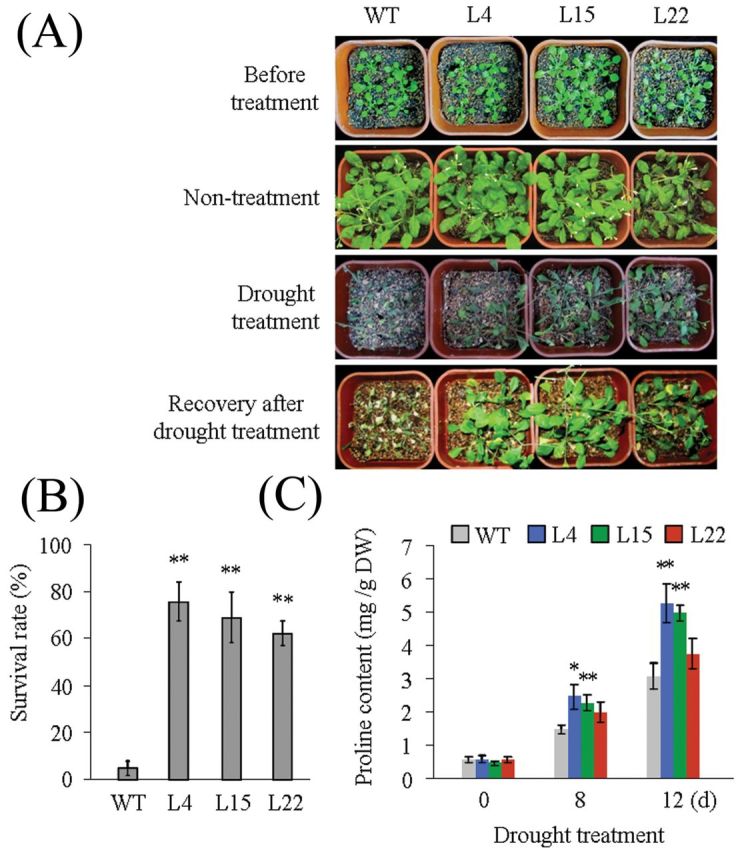
Roles of *SiARDP* in transgenic *Arabidopsis*. (A) Drought tolerance of WT and transgenic *Arabidopsis*. Three-week-old WT and transgenic *Arabidopsis* plants were not watered for 2 weeks, and then rewatered for 5 d. (B) Survival rate of plants in (A) under drought stress. This experiment had three replicates, and each experiment comprised at least 30 plants. (C) Proline content in WT and transgenic plants after drought stress. Each data point had three replicates. For B and C, the error bars indicate ±SD, and * and ** indicate statistically significant differences at *P* < 0.05 and *P* < 0.01 (Student’s t-test), respectively. This figure is available in colour at *JXB* online.

To test the effects of *SiARDP* overexpression on drought tolerance, 2-week-old transgenic and WT plants growing on soil were not watered for 2 weeks, and then rewatered and grown under normal conditions for 5 d. Most of the transgenic *Arabidopsis* resumed growing but the WT did not (Fig. 5A). The survival rates of the three transgenic lines were greater than 58%. In contrast, only ~5% of the WT plants survived (Fig. 5B). The content of free proline was higher in transgenic than in non-transgenic *Arabidopsis* under long-term drought stress (Fig. 5C). However, we did not find obvious differences between the transgenic and non-transgenic plants under the low temperature treatment (data not shown).

Together, these results indicated that the overexpression of *SiARDP* improves tolerance of drought and high salinity stress in transgenic *Arabidopsis*.

### *SiARDP* overexpression enhances the drought tolerance of foxtail millet

**Fig. 6.**
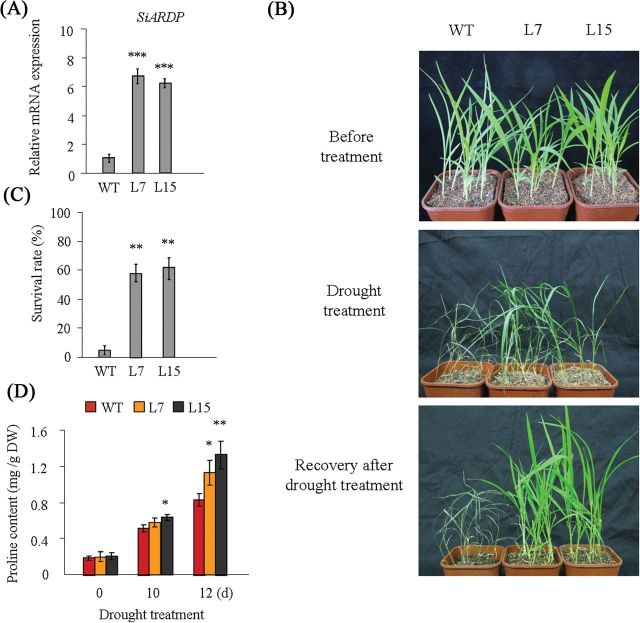
Roles of *SiARDP* in transgenic foxtail millet. (A) Expression levels of *SiARDP* in WT and two transgenic lines, L7 and L15, by qRT-PCR. (B) Drought tolerance of WT and transgenic millet. Two-week-old millet seedlings were not watered for 14 d, then rewatered for 5 d and phenotypes observed. (C) Survival rate of plants in (B) under drought stress. This experiment had three replicates, and each experiment comprised at least 36 plants. (D) Proline content in WT and transgenic plants after drought stress. Each data point had three replicates. Error bars indicate ±SD, and *, **, and *** indicate statistically significant differences at *P* < 0.05, *P* < 0.01, and *P* < 0.001 (Student’s t-test), respectively. This figure is available in colour at *JXB* online.

To further analyse the function of *SiARDP*, it was transformed into foxtail millet under the control of the ubiquitin promoter. Based on the results of qRT-PCR (Fig. 6A), two overexpressing T2 transgenic lines were selected for further research. Two-week-old WT and transgenic millet seedlings were grown under drought stress conditions for 14 d, and then watered and grown under normal conditions for 5 d. The survival rate of transgenic millet seedlings was higher than that of the WT (Fig. 6B and C). When water was withheld for 10 and 12 d, the free proline content of WT and transgenic millet was examined. The results showed that more proline accumulated in transgenic millet seedlings than in WT seedlings under drought stress (Fig. 6D). However, there were no obvious differences between WT and transgenic plants under the high salinity treatment (data not shown).

### *SiARDP* regulates stress-responsive gene expression

**Fig. 7.**
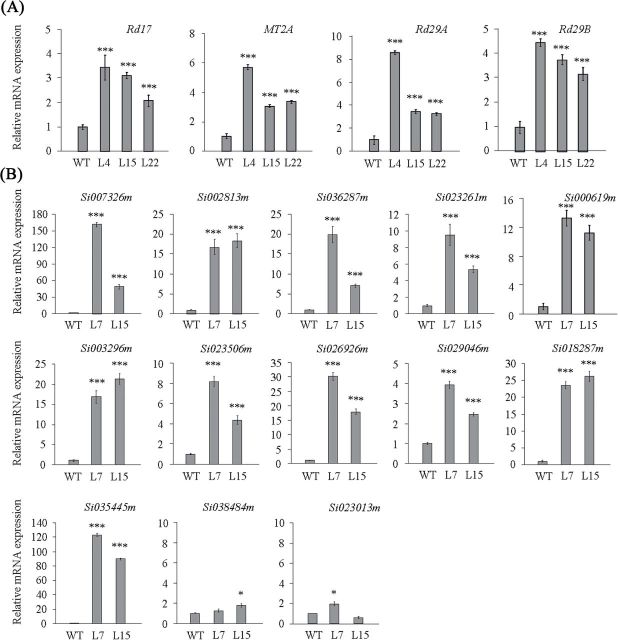
*SiARDP* regulates stress-responsive gene expression levels in transgenic *Arabidopsis* and transgenic foxtail millet. (A) Stress signal-relevant gene expression levels were observed in *SiARDP* transgenic *Arabidopsis* lines L4, L15, and L22 by qRT-PCR. *UBQ5* (At3g62250) was amplified as a normalization control. (B) Stress relevant gene expression levels were observed in *SiARDP* transgenic foxtail millet lines L7 and L15 by qRT-PCR. Error bars indicate ±SD, and * and *** indicate statistically significant differences at *P* < 0.05 and *P* < 0.001 (Student’s t-test).

The transgenic *Arabidopsis* expressing *SiARDP* showed a higher tolerance to abiotic stress compared with the non-transgenic *Arabidopsis*. To analyse the expression of stress responsive genes in the transgenic *Arabidopsis*, four stress-relevant genes were chosen. Without stress treatment, the expression of *rd29A* and *MT2A* increased to significantly higher levels in the transgenic *Arabidopsis*, especially in line 4, than in WT (Fig. 7A), and *Rd29B* and *Rd17* showed slightly higher transcript levels in *SiARDP*-transgenic *Arabidopsis* lines than in control lines. These results imply that *SiARDP* may regulate stress tolerance genes in transgenic *Arabidopsis*.

We chose 13 stress-relevant genes from foxtail millet to analyse the function of *SiARDP* and to study the relationship between stress tolerance and gene expression. The 13 genes were categorized according to putative gene functions. qRT-PCR analysis was used to examine changes in the expression levels of these genes. The expression of four LEA genes, *Si007326m*, *Si002813m*, *Si036287m*, and *Si023261m*, four genes encoding dehydrins, *Si003296m*, *Si023506m*, *Si026926m*, and *Si029046m*, and three other drought stress relevant genes, *Si000619m*, *Si018287m*, and *Si035445m*, increased in transgenic foxtail millet plants under normal conditions (Fig. 7B). Additionally, we found that the expression levels of two salt and low temperature tolerance-relevant genes, *Si023013m* and *Si038484m*, were not obviously altered in transgenic foxtail millet (Fig. 7B). There was at least one DRE core element in the 1000-bp promoter region of these 13 genes (Supplementary Table S1 at JXB online). These results indicate that *SiARDP* may activate the expression levels of the drought stress-relevant genes through direct binding to the DRE core element in their promoter regions.

### Identification of *SiAREB1* and *SiAREB2*

To further study the stress signal transduction pathway mediated by *SiARDP* in foxtail millet, we analysed the promoter of *SiARDP* and found two ABRE motifs (Supplementary Figure S2 at JXB online). The AREB/ABF subfamily binds to the ABRE core sequence, and *SiARDP* might be regulated by AREB-type transcription factors in foxtail millet. We found six putative AREB-type genes in foxtail millet. Among these genes, two multiple stress-inducible genes, named *SiAREB1* and *SiAREB2*, were chosen for further research. *SiAREB1* encoded a protein of 357 amino acids, and *SiAREB2* encoded a protein of 280 amino acids. Both proteins had a basic leucine zipper (bZIP) domain. *SiAREB1* harbours three N-terminal and one C-terminal conserved domains, and *SiAREB2* has two N-terminal and one C-terminal conserved domains.

The expression levels of *SiAREB1* and *SiAREB2* were induced by dehydration, high salinity, and ABA treatments, but they were not affected by cold stress (Supplementary Figure S3A at JXB online). Both *SiAREB1* and *SiAREB2* were highly expressed in leaf tissue (Supplementary Figure S3B at JXB online). A subcellular localization assay showed that both SiAREB1 and SiAREB2 fused to GFP were located in the nucleus of the foxtail millet protoplast (Fig. 3F–L). The abilities of SiAREB1 and SiAREB2 to activate transcription were examined using a yeast transcriptional activation system. Yeast cells separately transformed with pBD-SiAREB1 and pBD-SiAREB2 were grown on medium lacking threonine and histidine. SiAREB1 and SiAREB2 strongly activated β-galactosidase compared with the negative control (Supplementary Figure S4 at JXB online). These results suggest that SiAREB1 and SiAREB2 are AREB-type transcription factors associated with abiotic stress in foxtail millet.

### SiAREB1 and SiAREB2 bind to the ABRE motifs in the promoter of *SiARDP*

**Fig. 8.**
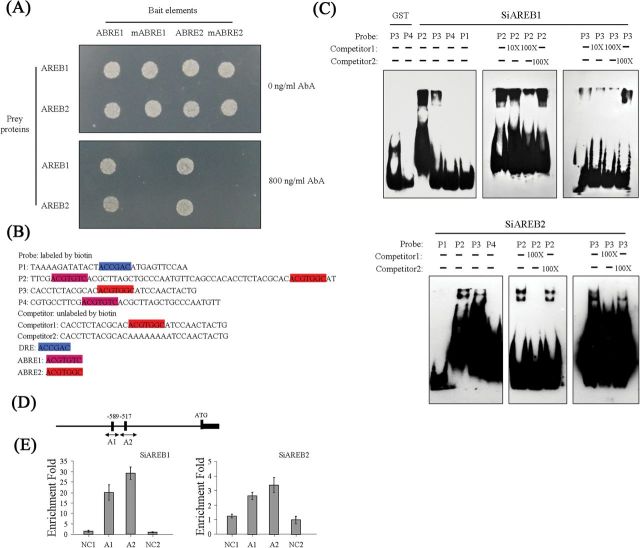
DNA-binding ability of SiAREB1 and SiAREB2. (A) Yeast one-hybrid assay in which SiAREB1 and SiAREB2 bind to ABRE1 and ABRE2. The bait sequences are shown in Supplementary Table S2 at *JXB* online. (B) SiAREB1 and SiAREB2 bind to various elements. The DRE (highlighted in P1), AREB1 (highlighted in P2 and P4) and AREB2 (highlighted in P2, P3, and Competitor1) probe sequences are labelled. (C) SiAREB1 and SiAREB2 binding to the elements is shown in the presence of changing concentrations of competitors. (D) Schematic diagrams showing the 1kb promoter sequence of *SiARDP*. A1 and A2 show the putative AREB binding sites, and ATG is the translation start site at position +1. ChIP-qPCR target regions are indicated by arrows. (E) ChIP-qPCR assay of SiAREB1 and SiAREB2 binding to the *SiARDP* promoter. An upstream region of the SiARDP promoter is amplified as a negative control (NC1), and a coding region of SiARDP is amplified as another negative control (NC2). The primers used for the ChIP-qPCR are listed in Supplementary Table S2 at *JXB* online. Error bars indicate ±SD. At least three independent experiments were performed with similar results. This figure is available in colour at *JXB* online.

To examine whether *SiAREB1* and *SiAREB2* are involved in the regulation of SiARDP, a yeast one-hybrid assay was performed. Two bait sequences were inserted into a bait plasmid. One has three 17-bp sequences containing ABER1 (ACGTGTC) and another has three 17-bp sequences containing ABRE2 (ACGTGGC). Both sequences were in the *SiARDP* promoter. Meanwhile, mutant bait constructs containing AAAAAAA as a substitute for ABRE1 and ABRE2 core elements were also created. The bait strains and mutant bait strains grew on media lacking uracil, but did not grow on media containing 800ng ml^−1^ AbA (data not shown). *SiAREB1* and *SiAREB2* were independently inserted into the expression vector pGADT7-AD. Then, the two vectors were transformed separately into the bait strains or mutant bait strains. The bait yeast cells transformed with pGADT7-*SiAREB1* or pGADT7-*SiAREB2* grew on media lacking leucine and containing 800ng ml^−1^ AbA, but the transformed mutant bait yeast cells did not (Fig. 8A).

To confirm the results of the yeast one-hybrid assay, an EMSA was performed. The DRE core element ACCGAC in the *rd29A* promoter was used as probe 1 (P1) (Fig. 8B). The sequence that contained two ABRE elements in the promoter of *SiARDP* was used as probe 2 (P2). The sequence that contained ABRE2 and that contained ABRE1 in the *SiARDP* promoter were used as probe 3 (P3) and probe 4 (P4), respectively (Fig. 8B). Full-length *SiAREB1* and *SiAREB2* were expressed as glutathione *S*-transferase (GST) fusion proteins in *E. coli*. We examined whether SiAREB1 and SiAREB2 could bind to these probes. Both SiAREB1 and SiAREB2 had the ability to bind to P2 and P3 but not P4 (Fig. 8C). Both SiAREB1 and SiAREB2 proteins bound efficiently to P2, while the binding affinities of SiAREB1 and SiAREB2 for P3 were weaker. With the addition of the unlabelled core probe, the signal was reduced, but the addition of the mutant probe did not interfere with binding. These results indicate that SiAREB1 and SiAREB2 specifically bind to the ABRE2 element in the promoter of *SiARDP*.

Additionally, to analyse whether SiAREB1 and SiAREB2 could bind to the promoter of *SiARDP in vivo*, a transient ChIP-qPCR was performed on WT foxtail millet protoplasts. A sequence analysis showed that two ABRE elements, A1 and A2, existed in a 1-kb region upstream of the translation start site (Fig. 8D). qRT-PCR was used to detect the results of the ChIP analysis. As shown in Fig. 8E, the transient expression levels of the SiAREB1-GFP and SiAREB2-GFP fusion proteins indicated that they could bind to the *SiARDP* promoter. The SiAREB1 protein’s interaction with the *SiARDP* promoter was stronger than the interaction of the SiAREB2 protein. These results indicated that SiAREB1 and SiAREB2 directly regulate *SiARDP in vivo*, and that SiAREB1 may be the main regulatory factor.

## Discussion

**Fig. 9.**
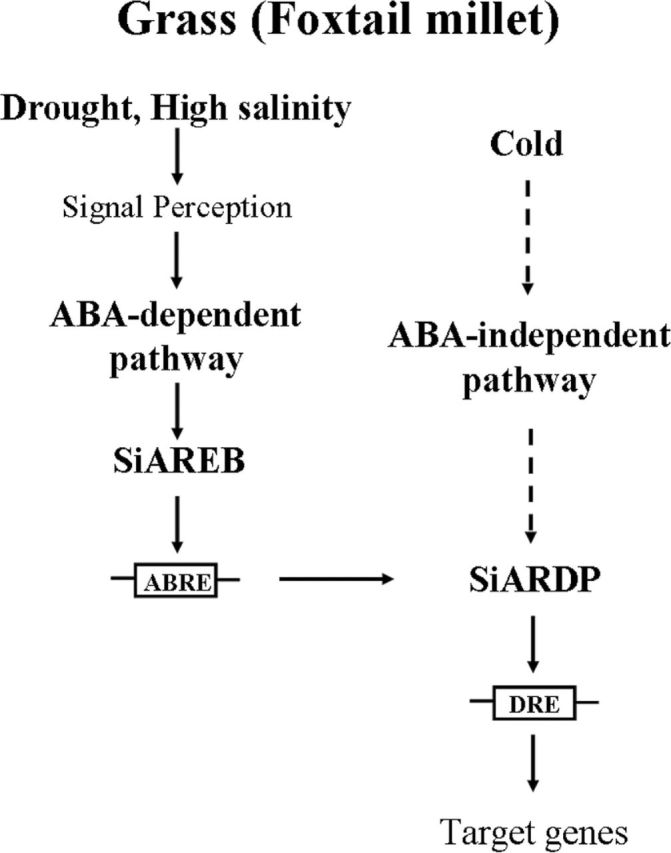
Model of the *SiARDP* response to abiotic stress in foxtail millet. *SiARDP* is regulated by SiAREB transcription factors in the ABA-dependent pathway under drought and high salinity stress. Under cold stress, *SiARDP* may be involved in the ABA-independent pathway.

The regulatory networks of abiotic stress are complicated. ABA signalling plays an important role in plants that are under abiotic stress, such as drought conditions (Busk and Pagès, 1998; Rock, 2000; Yamaguchi-Shinozaki and Shinozaki, 2006). AREB proteins are very important transcription factors in the ABA-responsive signal pathway, and their activation of AREB transcription factors is necessary for ABA-dependent phosphorylation (Johnson *et al.*, 2002; Kobayashi *et al.*, 2005; Furihata *et al.*, 2006). In previous research, the AREB and DREB transcription factors were reported to belong to the ABA-dependent and ABA-independent signal pathways, respectively. However, a few of the DREB-type transcription factors were found to be involved in the ABA-dependent pathway (Egawa *et al.*, 2006). In the present study, the transcript level of *SiARDP* was upregulated not only by dehydration, high salinity, and low temperature treatments, but also by exogenous ABA. We found two ABRE core elements, ABRE1 and ABRE2, in the promoter of *SiARDP*. Two AREB proteins, named *SiAREB1* and *SiAREB2*, were identified as AREB transcription factors and confirmed to bind to ABRE2 (Fig. 8C and E). The transcript levels of *SiAREB1* and *SiAREB2* increased, similarly to the level of *SiARDP*, under dehydration, salt, and ABA treatments, but not under the low temperature treatment (Supplementary Figure S3A at JXB online). In addition, three genes, *SiARDP*, *SiAREB1*, and *SiAREB2*, exhibited the same tissue-specific expression patterns in foxtail millet (Fig. 2B and Supplementary Figure S3B at JXB online). The co-expression of these three genes in the same tissues at the same time indicated that *SiAREB1* and *SiAREB2* may be involved in the regulation of *SiARDP*. These results suggest that *SiARDP* is a member of two abiotic stress signal transduction pathways. One is the ABA-dependent signal pathway for drought and salt stress regulated by *SiAREB1* and *SiAREB2*, and the other appears to be an ABA-independent pathway for low temperature stress regulated by other transcription factors (Fig. 9).

ABRE is a major *cis*-acting element in the ABA-dependent signalling pathway. Most of the ABA-inducible genes contain ABRE elements in their promoters, and a single ABRE element is not enough for ABA-dependent transcription (Shen *et al.*, 1996; Hobo *et al.* 1999). SiAREB1 and SiAREB2 specifically bind to P3, which contains an ABRE2 element, but not to P4, which contains ABRE1 (Fig. 8B and C). Analysis of the P3 sequence found that a G-box-like element exists in the sequence, while a similar element does not exist in the P4 sequence. The results of the yeast one-hybrid assay showed that SiAREB1 and SiAREB2 bind to the ABRE1 element. This is probably because the triplicate 17-bp sequence improves the affinity of SiAREB1 and SiAREB2 proteins to the ABRE1 element. Furthermore, the ChIP-qPCR assay showed that SiAREB1 and SiAREB2 bind to both A1 (containing the AREB1 element) and A2 (containing the ABRE2 element) in the promoter region of *SiARDP* (Fig. 8D and E) *in vivo*. There are ~72bp between A1 and A2, and this short sequence could not be sheared completely by sonication. This may be the main reason why A1 and A2 were enriched in the ChIP-qPCR assay. The results imply that SiAREB1 and SiAREB2 can bind to the promoter of *SiARDP* and that the peripheral sequences of the ABRE element are very important to AREB transcription factor binding.

The DREB proteins are very important transcription factors in abiotic stress signal transduction pathways in plants. The stability of the DREB2 proteins is very important to their function in *Arabidopsis*. The full-length DREB2A protein was unstable in the nucleus and was degraded by the ubiquitin-proteasome pathway (Qin *et al.*, 2008), while the DREB2A-CA (constitutive active form) with a deleted negative regulatory domain showed stable expression in the nucleus and upregulated some stress-induced genes (Sakuma *et al.*, 2006). Some of the DREB2 proteins that do not contain the negative regulatory domain, such as ZmDREB2A, OsDREB2B, and PeDREB2 (Qin *et al.*, 2007; Chen *et al.*, 2009; Matsukura *et al.*, 2010), enhance drought resistance in transgenic plants. SiARDP, reported here, also did not contain the negative regulatory region and is stable in the nucleus. Overexpression of *SiDREB2* improved both drought and high salinity stress tolerance in transgenic *Arabidopsis*, and the results were consistent with the expression patterns of *SiARDP* under dehydration and salt stress (Figs 2A, 4B and D, and 5A). However, the overexpression of *SiARDP* only enhanced drought tolerance in transgenic millet (Fig. 6B). Similarly, *DREB2C* responds to salt, mannitol, and cold, but overexpression of *DREB2C* only improves thermotolerance in *Arabidopsis* (Lim *et al.*, 2007). The expression level of *ZmDREB2A* was increased by drought, salt, cold, and heat in maize, while overexpressing it enhanced drought tolerance and thermotolerance in transgenic *Arabidopsis*. These studies suggest that DREB2 proteins have different functions in different plants, and heterologous expression may be an important reason.

The DREB genes enhance stress tolerance by regulating their target genes. Overexpressing *SiARDP* induced the expression of drought and salt stress-relevant genes (*Rd17*, *MT2A*, *Rd29A*, and *Rd29B*) in transgenic *Arabidopsis* (Fig. 7A). In a previous study, *Rd17*, *Rd29A* and *Rd29B* were induced by drought and salt stress, and *MT2A* was induced by drought stress. In transgenic millet most of the induced genes were related to drought stress. Although the precise functions of these induced genes in the transgenic foxtail millet are still unknown, previous studies have implied that these proteins, especially LEA, play a role in protecting the cells from the irreversibly damaging effects of a water deficit (Ingram and Bartels, 1996; Thomashow, 1999; Zhu, 2001). Meanwhile, two salt and low temperature stress-relevant genes, *Si038484m* and *Si023013m*, were not induced. The target genes of *SiARDP* in transgenic *Arabidopsis* and transgenic millet are different, and there is a positive relationship between the stress induction of genes and stress tolerance. These results indicate that *SiARDP* is involved in the drought stress signalling pathway in foxtail millet.

Plant abiotic stress tolerance involves complex physiological and biochemical processes. In these processes, transcription factors are important to the plant’s ability to adapt to stresses. In the present study, we focused on how *SiARDP* was involved in the ABA-responsive signalling pathway, as well as the ability of *SiARDP* to increase abiotic stress tolerance in plants. Based on our study, we propose a regulatory role for SiAREB transcription factors in regulating the expression of *SiARDP* under drought and salt stress (Fig. 9). Our findings show that SiAREB1 and SiAREB2 bind to the ABRE elements in the promoter region of *SiARDP* and activate the expression of SiARDP, and that the target genes of *SiARDP* are then activated in response to drought and salt stress.

## Supplementary material

Supplementary data can be found at *JXB* online.

Supplementary Table S1. Stress-relevant genes in overexpressing foxtail millet SiARDP plants.

Supplementary Table S2. Primers and bait sequences used in this study.

Supplementary Figure S1. Comparison of amino acid sequences and phylogenetic analysis of the foxtail millet SiARDP with other DREB proteins.

Supplementary Figure S2. ABREs identified in the SiARDP promoter.

Supplementary Figure S3. Expression pattern assay of *SiAREB1* and *SiAREB2*.

Supplementary Figure S4. Transcriptional activation assay of SiAREB1 and SiAREB2.

## Funding

This work was supported by the National Basic Research Programme of China (2012CB215301) and the National Science Foundation of China (grant No. J1103520).

## Supplementary Material

Supplementary Data
