# Supplementary material for: An ABA-responsive DRE-binding protein gene from Setaria italica, SiARDP, the target gene of SiAREB, plays a critical role under drought stress
Source: J Exp Bot. 2014 Jul 28;65(18):5415–27. doi: 10.1093/jxb/eru302 (PMC4157718; doi:10.1093/jxb/eru302)
Supplement: Supplementary Data [file supp_65_18_5415__index.html]

An ABA-responsive DRE-binding protein gene from Setaria italica, SiARDP, the target gene of SiAREB, plays a critical role under drought stress — An ABA-responsive DRE-binding protein gene from Setaria italica, SiARDP, the target gene of SiAREB, plays a critical role under drought stress — Supplementary Data 

# An ABA-responsive DRE-binding protein gene from *Setaria italica*, *SiARDP*, the target gene of SiAREB, plays a critical role under drought stress

## Supplementary Data

Data files

**Files in this Data Supplement:**

- Supplementary Data - Supplementary Data
