# Supplementary material for: An ABA-responsive DRE-binding protein gene from Setaria italica, SiARDP, the target gene of SiAREB, plays a critical role under drought stress
Source: J Exp Bot. 2014 Jul 28;65(18):5415–27. doi: 10.1093/jxb/eru302 (PMC4157718; doi:10.1093/jxb/eru302)
Supplement: Supplementary Data [file supp_eru302_jexbot127811_file001.pdf]

An ABA responsive DRE-binding protein gene from *Setaria italica, SiARDP,* the target gene of SiAREB, plays critical role under drought stresses. *Cong Li, Jing Yue, Xiaowei Wu, Cong Xu, Jingjuan Yu*

SUPPLEMENTARY DATA

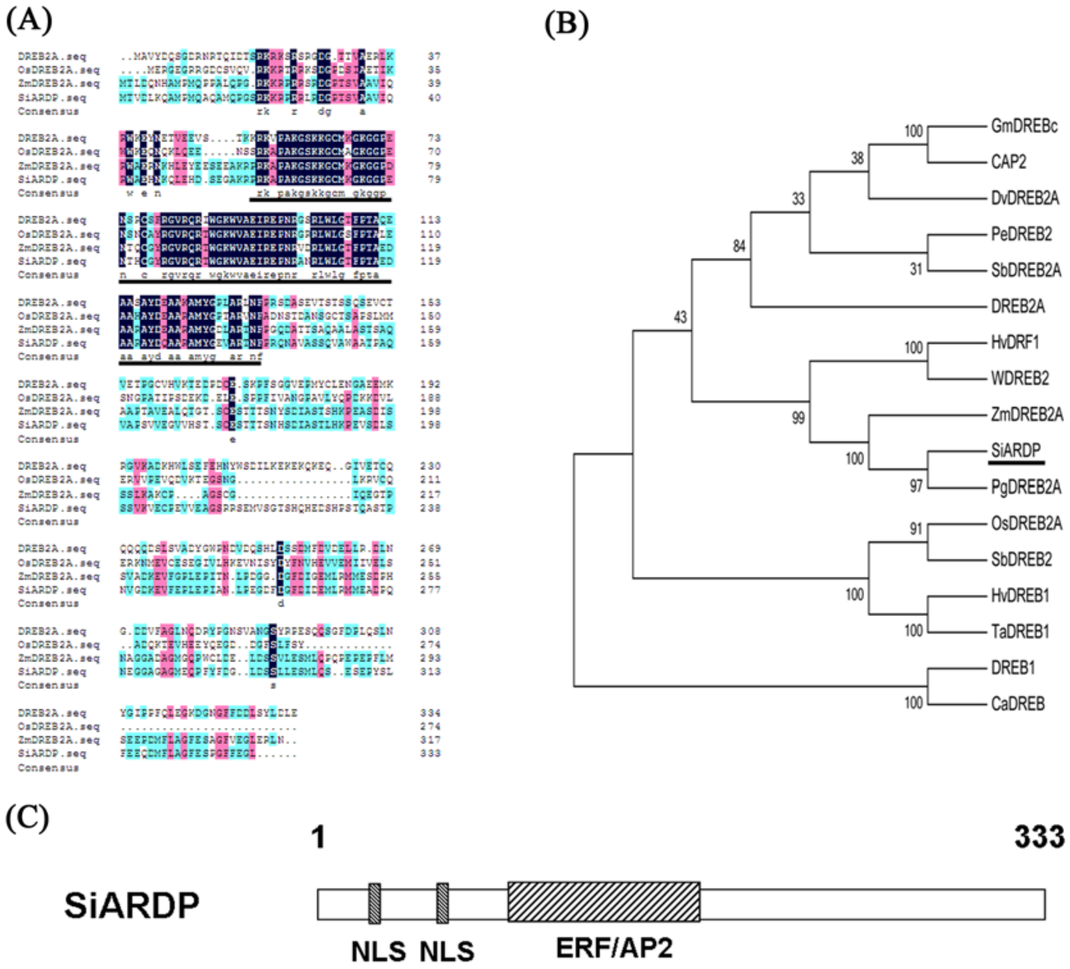

Figure S1. Comparison of amino acid sequences and phylogenetic analysis of the *Setaria italica* ABA-responsive DREB-like protein (*SiARDP*) with other dehydration responsive element binding proteins (DREB). (A) The entire protein sequences DREB2A, ZmDREB2A, OsDREB2A and SiARDP were analyzed. The ERF/AP2 DNA-binding domains are underlined. (B) A phylogenetic tree of the DREBs protein from different species. The multiple alignments were generated by MUSCLE and the phylogenetic tree was constructed by MEGA5.2.2 using a bootstrap test of phylogeny and the Neighbor Joining test with default parameters. The accession unmber of proteins are: ZmDREB2A (AB218832), PgDREB2A (DQ227697), SiARDP, HvDRF1 (AY223807), WDREB2 (HQ171443), HvDREB1 (DQ012941), TaDREB1 (DQ195068), OsDREB2A (AF300971), SbDREB2 (JF915841), SbDREB2A (GU809211), DREB2A (AB007790), DvDREB2A (EF633987), CAP2 (HSU02390), GmDREBc (AAP83131), PeDREB2 (EF137176), CaDREB (AY496155) and DREB1 (JN866912). (C) Schematic diagrams of the SiARDP protein. NLS, nuclear localization signal: ERF/AP2, ERF/AP2 DNA binding domain.

TGCTACAGTTGCGCATTTATGGTAAAATCGCAACTTTAACACTTCCAAATTGGTAAC
TAAGCAAGGTCTGAATATCTCAAAGACGGGCCATCGTGCCTTCGACGTGTCACGCT
TAGCTGCCCAATGTTCAGCCACACCTCTACGCACACGTGGCATCCAACTACTGGCC
AGACTTAAAATAGAGACGGAATCCATTTAGAGGCCGGACTTTAATAACCACAGGA
GGAAAAATCCACTACCGAAGGAAAGTGCAGCGACTGGGGCTGATCAAGTTTGCTTA
GGCGCCAAGTCTCTCGATCCTTCCTTGCAAAGGCGCACGACCGGTCCAGCGCACGC
CTTACGTCACCTCGCCCCTCCCTGTTCACCTCACCGCACCCGGCTCACGTGAACCCC
TCCCTCTGACGGGCGGGCCCCAGCACAGACGAACCCCGTGGACCGGCCGCGAGCC
ACCAGAATACGAGATATTTTCCCGTTGGGTGGGGCCCACGGAGCGCGTCGAGGCAG
GCCTATAAATAGCCCGCGCACGCCCTCCCATCGGACTTCCGATTTCTCTCTCTTCTTT
CCCCCACACATCGACTTTTCTTCTCG

ABRE1: ACGTGTC

ABRE2: ACGTGGC

TATA box: TATAAAT

Figure S2. ABRE *cis*-elements identified in the *Setaria italica* ABA-responsive DREB-like protein (SiARDP) promoter. The ABRE1 (purple bar), ABRE2 (red bar) and TATA box (blue bar) are shown.

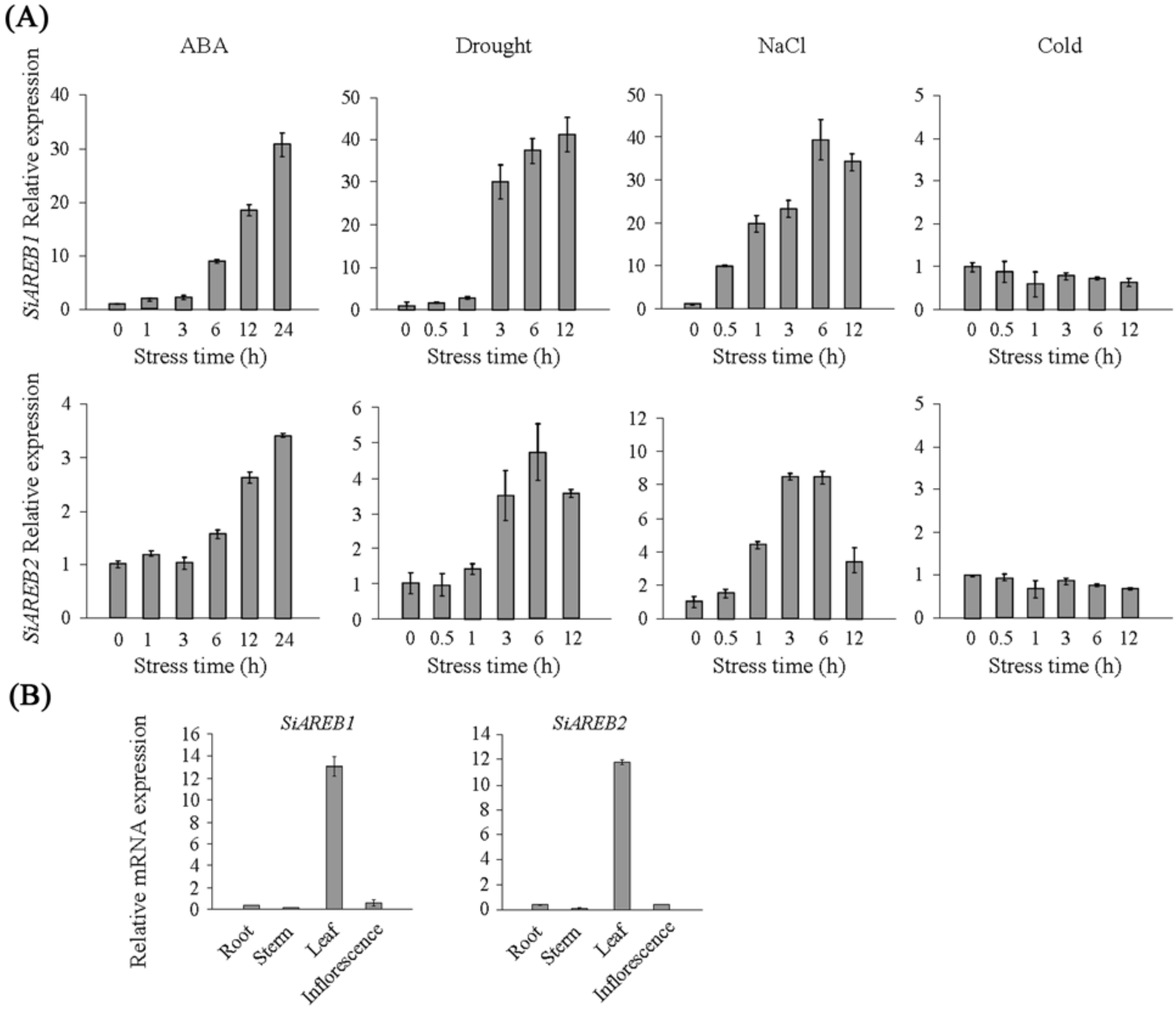

Figure S3. Expression pattern assay of *Setaria italica* ABA-responsive element binding genes *SiAREB1* and *SiAREB2*. (A) Transcript levels of *SiAREB1* and *SiAREB2* in response to various stresses in millet seedling as demonstrated by qRT-PCR. The millet seedlings were treatment under NaCl (100 mM), PEG (20% V/V), ABA (10 μM) and 4°C, respectively at selected time points. (B) Transcript levels of SiAREB1 and SiAREB2 in different parts of millet as demonstrated by qRT-PCR.

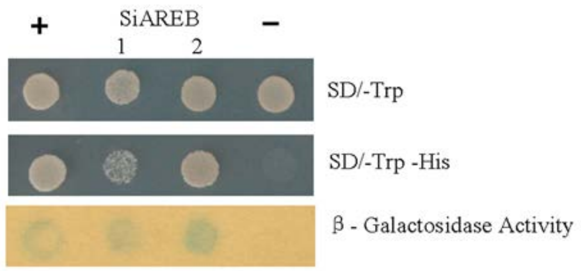

Figure S4. Transcriptional activation assay of *Setaria italica* ABA-responsive element binding-type proteins SiAREB1 and SiAREB2. + indicate the yeast transformed the pGAL4 plasmid as the positive control. - indicate the yeast transformed the pBD-GAL4 plasmid as the negative control.

Table S1. Selected stress-responsive genes in overexpression *SiARDP* millet plants

Gene name	Description	No. of DREs in promoter
Si007326m	Late embryogenesis abundant protein, group 1 protein	3
Si002813m	Late embryogenesis abundant protein (LEA) family protein	2
Si036287m	Late embryogenesis abundant domain-containing protein / LEA domain-containing protein	2
Si023261m	Late embryogenesis abundant protein (LEA) family protein	2
Si003296m	Dehydrin induced protein 1	1
Si023506m	Dehydration-induced protein (ERD15)	3
Si026926m	Dehydrin family protein	3
Si029046m	Early-responsive to dehydration stress protein (ERD4)	2
Si035445m	Glycosyl hydrolase superfamily protein	3
Si018287m	Remorin family protein	3
Si000619m	Heat shock protein 70	1
Si038484m	Low temperature and salt responsive protein family	5
Si023013m	Salt tolerance zinc finger	1

**Table S2.** Primers and bait sequences used in this study.		
Gene name	Forward primer	Reverse primer
Rd29A	5'-ACACCAGCAGCACCCAGAAGA-3'	5'-CGGAAGACACGACAGGAAACAC-3'
Rd29B	5'-CGCCTGTTACGCCATTGTCA-3'	5'-GCTCCACGGTGTAAGCCTGTTT-3'
Rd17	5'-CCAGGTTATCATGCCAAGACCA-3'	5'-AATGCAATCAACGAAAGCCACA-3
MT2A	5'-TGGAGGAAACTGCGGATGTG-3'	5'-TCCCCTGAAGCCTCGTACTGA-3'
UBQ5	5'-CTCCTTCTTTCTGGTAAACGT-3'	5'-GGTGCTAAGAAGAGGAAGAAT-3'
Si007326m	5'-GAGCAGGATGCAGGCTGTGA-3'	5'-AAGTTAAGCGAGCGACCCAAA-3'
Si002813m	5'-CAACGACGGCACCACCAAT-3'	5'-TCAAAGCAAAGTGAAACGCACA-3'
Si036287m	5'-TCGGAGGACGAGCTGTGAGG-3'	5'-CACGATGGCTGGAACAAAGAGT-3'
Si023261m	5'-CGGCAACACCAACAAGGACTC-3'	5'-CGCCAAACTTACAGCACCAAAT-3'
Si003296m	5'-CCACGCCACCAACCAAGC-3'	5'-CTTTTCTCCGGTGCCCTCC-3'
Si023506m	5'-CCTCGCTGAAGGACCGATACA-3'	5'-AACCAAAACAACGCAAGGCTAA-3'
Si026926m	5'-ATGGGTGGGAGGAGGAAGAAG-3'	5'-CGGAACGCCGTGACAGGTA-3'
Si029046m	5'-GCACCAGGGTTCCAAATGACA-3'	5'-TGTTGGCGAATGCTGGGTAA-3'
Si035445m	5'-CCGTCTACTTCAGCGGGTTCA-3'	5'-GCCGAAGTCGTGGAAGTTGTT-3'
Si018287m	5'-ACGCCCTGAAAGCGACCA-3'	5'-AAGCCACAAGCACGCACATC-3'
Si000619m	5'-ACCAGGTCGCCATGAACCC-3'	5'-TGATTGTGGAGCCGAGGTAGG-3'
Si038484m	5'-TGCTGCCGAAGACTAGACCAAA-3'	5'-ACACCACGCCCCATAACCC-3'
Si023013m	5'-GCGGCTTCGACCTCAACCT-3'	5'-AAACCAATTCCACCAATCACCA-3'
SiARDP	5'-CAAGTTGCTTGGGCGGCAACC-3'	5'-ACGTGAACCAGCCTCCACAAC-3'
SiAREB1	5'-GGAGATCACGCTGGAGGAGT-3'	5'-AGGCATCAATGGAGCAAACA-3'
SiAREB2	5'-ACGCTCGGCGAGATGACT-3'	5'-ATTCCCAAGAAAGGCGGTAC-3'
Siactin	5'-GTGCTTTCCCTCTACGCCAGTG-3'	5'-ACCGCTGAGCACAATGTTACCA-3'
NC1	5'-ATGGATAAGCCTTGAGGGTA-3'	5'-CCTACCGGCATCAAATGATG-3'
A1	5'-TCGCAACTTTAACACTTCCA-3'	5'-ATTGGGCAGCTAAGCGTGAC-3'
A2	5'-CCACACCTCTACGCACACGT-3'	5'-CACTTTCCTTCGGTAGTGGA-3'
NC2	5'-CAAGTTGCTTGGGCGGCAACC-3'	5'-ACGTGAACCAGCCTCCACAAC-3'
Bait sequences		
ABRE1	5'-AGCTTTTCGACGTGTCACGCTTCGAC GTGTCACGCTTCGACGTGTCACGCC-3'	5'-TCGAGGCGTGACACGTCGAAGCGTGACA CGTCGAAGCGTGACACGTCGAAA-3'
ABRE2	5'-AGCTTGCACACGTGGCATCCGCACAC GTGGCATCCGCACACGTGGCATCCC-3'	5'-TCGAGGGATGCCACGTGTGCGGATGCCA CGTGTGCGGATGCCACGTGTGCA-3'
mABRE1	5'-AGCTTTTCGAAAAAAAAACGCTTCGA AAAAAAACGCTTCGAAAAAAAAACGCC-3'	5'-TCGAGGCGTTTTTTTTCGAAGCGTTTTTT TTCGAAGCGTTTTTTTTCGAAA-3'
mABRE2	5'-AGCTTGCACAAAAAAAAATCCGCACA AAAAAAATCCGCACAAAAAAAAATCCC-3	5'-TCGAGGGATTTTTTTTGTGCGGATTTTTTT TGTGCGGATTTTTTTTGTGCA-3'
